# SKLB023 hinders renal interstitial fibrosis in obstructive nephropathy by interfering TGF-β1/Smad3 signaling†

**DOI:** 10.1039/c8ra00018b

**Published:** 2018-02-06

**Authors:** Yanhuan Feng, Jun Xu, Fan Guo, Rongshuang Huang, Min Shi, Lingzhi Li, Liang Ma, Ping Fu

**Affiliations:** Kidney Research Institute, Division of Nephrology, West China Hospital of Sichuan University No. 37 Guoxue Allay Chengdu 610041 China liang_m@scu.edu.cn fupinghx@scu.edu.cn +86 28 85423341 +86 28 85164167

## Abstract

Renal fibrosis is the principal process underlying the progression of chronic kidney disease to end-stage renal disease. It is a relatively uniform response involving glomerulosclerosis, tubulointerstitial fibrosis and changes in renal vasculature. A considerable number of studies have confirmed that inducible nitric oxide synthase (iNOS) was highly expressed in renal interstitial fibrosis and the overexpression of iNOS played a negative role in kidney disease progression. In our previous study, SKLB023 as a novel small-molecule inhibitor of iNOS, blocked joint inflammation and cartilage destruction in arthritis. However, the pharmacological role and function of SKLB023 in renal fibrosis remained poorly understood. In the study, oral administration of SKLB023 (25 and 50 mg per kg per day) for 7 day exhibited potent anti-fibrotic effects against the model UUO using the pathological assessment of H & E and Masson's trichrome staining. SKLB023 inhibited the expression of α-SMA, col I, col IV, fibronectin and further decreased iNOS expression as well as TGF-β1/Smad3 phosphorylation in the injured kidney tissues of UUO mice. Similarly, SKLB023 suppressed *in vitro* features of fibrosis in TGF-β1-induced NRK-49F by the inhibition of the corresponding fibrotic protein expression. These findings confirmed that SKLB023 hindered renal interstitial fibrosis by interfering with TGF-β1/Smad3 signaling, highlighting that SKLB023 has potential in therapeutic strategies.

## Abstract 2

The novel small-molecule inhibitor of iNOS (SKLB023) hindered renal interstitial fibrosis *in vivo* and *in vitro* by interfering with TGF-β1/Smad3 signaling, highlighting that SKLB023 has potential in the therapeutic strategy for renal fibrosis.

## Graphical abstract

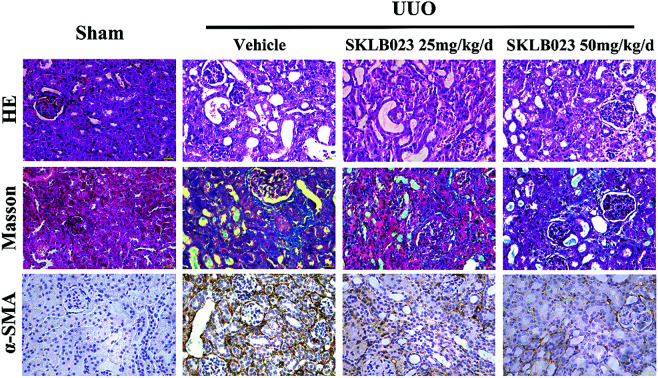

## Introduction

Renal interstitial fibrosis is the final common pathway leading to end-stage renal disease (ESRD).^1–5^ The main pathological features of renal interstitial fibrosis included the infiltration of inflammatory cells, excessive accumulation of extracellular matrix (ECM), tubular atrophy, and proliferation of fibroblasts. Although many fibrogenic factors regulated the renal interstitial fibrosis process, transforming growth factor-β (TGF-β)/Smads signaling is the central pathway.^6–8^ Many studies have established that TGF-β regulates biological responses in addition to fibrosis, such as cell proliferation, apoptosis, differentiation, autophage and immune response.^9,10^ Furthermore, these factors could be targeted to prevent the progression of fibrosis. Unfortunately, there is still a lack of renal interstitial fibrosis therapy regulating TGF-β or TGF-β/Smads signalling and it is very disappoint that there are no findings that could be translated from basic research into the clinic.

**Fig. 1:**
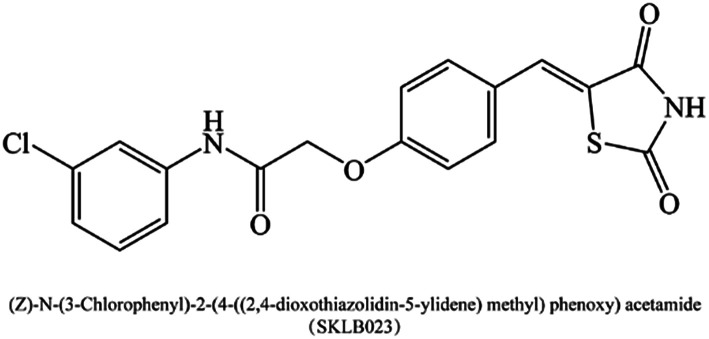
The chemical structure of SKLB023.

Previous studies confirmed that the fibrosis process was positively correlated with TGF-β level, as well as the abnormal expression of inducible nitric oxide synthase (iNOS).^11^ The iNOS is an enzyme that converts l-arginine and oxygen into l-citrulline and nitric oxide (NO) in a complex oxidoreductase reaction. The expression of iNOS is absent in resting cells but could be induced by immunological stimuli.^12^ When iNOS is up-regulated in response to stimulates, it generates 100–1000 fold more NO than does the other kind of NOS. The excessive NO production may exert detrimental effects under disease.^13^ In the liver fibrosis patients, a relative correlation was observed between iNOS and TGF-β1. Samples with low fibrosis displayed a faint positivity for iNOS and less TGF-β1 positive cells, while the expression of both proteins was increased in those with advanced fibrosis.^14,15^ The iNOS knockout mice fed a high-cholesterol diet for 6 weeks exhibited significant reductions in hepatic fibrosis and expression of TGF-β.^16^ Similarly, considerable studies also highlighted the importance of iNOS in chronic kidney disease.^17^ The iNOS-deficient and antibiotic-treated WT mice exerted less fibrotic, which stated that iNOS played a crucial role in the process of renal fibrosis.^18^

(*Z*)-*N*-(3-Chlorophenyl)-2-(4-((2,4-dioxothiazolidin-5-ylidene)methyl)phenoxy)acetamide (SKLB023, Fig. 1), is a novel small-molecule iNOS inhibitor based on thiazolidine-2,4-dione moiety.^19^ In our previous studies, SKLB023 was found to block joint inflammation and cartilage destruction of arthritis.^19^ However, the antifibrotic effects of SKLB023 in chronic kidney disease remain unclear. Thus, in the study, we chose the *in vivo* unilateral ureteric obstruction (UUO) mice and *in vitro* TGF-β1-induced NRK-49F cells to investigate the inhibition of SKLB023 against renal interstitial fibrosis and potential involved mechanism.

## Methods

### Animal model and treatment

The UUO model was established in male C57BL/6 mice (8–10 weeks old; 20–25 g) from the Animal Laboratory Center of Sichuan University (Chengdu, China). Briefly, the abdominal cavity was exposed *via* a midline incision and the left ureter was isolated and ligated. To examine the efficacy of SKLB023 in renal fibrosis after UUO injury, the mice were randomized into four groups (*n* = 6): (1) Sham operated mice, (2) UUO mice that received daily saline for 7 day by oral gavage (o.g.) after UUO, (3) UUO mice treated with SKLB023 25 mg per kg per day for 7 day by o.g. after UUO, (4) UUO mice treated with SKLB023 50 mg per kg per day for 7 day by o.g. after UUO. The mice were sacrificed and the kidneys were removed at day 7 for protein analysis and morphologic analyses. Experiments were approved by the Animal Ethics Committee of West China Hospital of Sichuan University (No. 2016-273).

### Morphologic and immunohistochemical analyses

Two-micrometer sections of paraffin-embedded kidney tissue were subjected to Masson's or HE staining using commercial kits (Sigma-Aldrich, USA) according to the manufacturer's protocol.^20^ Heat-induced epitope retrieval was performed on dewaxed slides in citrate buffer (pH 6.0) at 95 °C for 40 min. Then sections were exposed to peroxidase blocking solution (3% H_2_O_2_) prior to the addition of primary antibody, anti-α-SMA antibody (Abcam, USA) diluted to 1 : 500 in PBS. After incubation with primary antibody overnight at 4 °C, the slides were washed three times with PBS, and incubated with horseradish peroxidase (HRP)-incubated secondary antibody (Abcam, USA) for 45 min. The sections were washed again with PBS for three times. Subsequently, the slides were developed by diaminobenzidine (DAB) and counterstained with hematoxylin. Finally, the slides were observed under a light microscope.

Masson staining as well as immunostaining intensity were scored, and the scoring criteria were as follows: 10 high-power fields (×400) were randomly selected and photographed in each group. None, mild, moderate and severe involvement were scored as 0, 1, 2, or 3 according to the degree and extent of tubular degeneration and necrosis, tubular atrophy, inflammatory cell infiltration and fibrosis.^21^ The blue area of collagen by Masson staining, which represents the extent of the lesion, was calculated. The dyed area was measured by the average optical density in the immunostaining intensity scores.

### Cell culture and treatments

NRK-49F cells (American Type Culture Collection, USA) were cultured in phenol red-free Dulbecco's modified Eagle's medium DMEM (Hyclon, USA) supplemented with 5% FBS in a humidified atmosphere of 5% CO_2_ at 37 °C. The cells that reached approximately 50–70% confluence was used for *in vitro* experiments. To test the effect of SKLB023 on TGF-β1/Smad3 signaling, cells were serum starved by incubation with 0.5% FBS containing DMEM for 24 hours and then exposed to TGF-β1 (5 ng ml^−1^; R & D Systems, USA) and treated with SKLB023 for 48 hours. SKLB023 in cellular experiments was dissolved in DMSO (Sigma-Aldrich, USA).

### Western blot

Proteins were isolated from renal tissues or NRK-49F cells with a RIPA lysis buffer and then analyzed by Western blot. In brief, equal amounts of protein were separated by SDS-PAGE and then transferred on to a PVDF membrane (Bio-Rad, Hercules, CA, USA). Subsequently the membranes were incubated with primary antibodies against α-SMA (Abcam, USA), collagen I (Abcam, USA), collagen IV (Abcam, USA), fibronectin (Abcam, USA), iNOS (Abcam, USA), TGF-β1 (Abcam, USA), Smad3 (CST, USA), *p*-Smad3 (CST, USA) and GAPDH (Origene) or β-actin (Abcam, USA) overnight at 4 °C. Then the membranes were incubated with HRP-conjugated secondary antibodies (R & D systems) for 1 h at room temperature. Finally, the proteins on the membrane were developed with an enhanced chemiluminescencere agent (Millipore Corporation, Boston, MA, USA). The signals were detected using an Odyssey Infrared Imaging System (Bio-Rad, ChemiDoc MP, mANUSC, Bio-Rad Laboratories Inc., Hercules, CA, USA) and analyzed with the Image-Pro Plus program (Media Cybernetics, Inc.).

### Statistical analyses

Data are expressed as the mean ± SD. Comparisons between groups were made using one-way analysis of variance (ANOVA). Comparisons between two groups were conducted using the two-tailed *t* test. *P* < 0.05 was considered statistically significant.

## Results

### SKLB023 ameliorates renal interstitial fibrosis in UUO

**Fig. 2:**
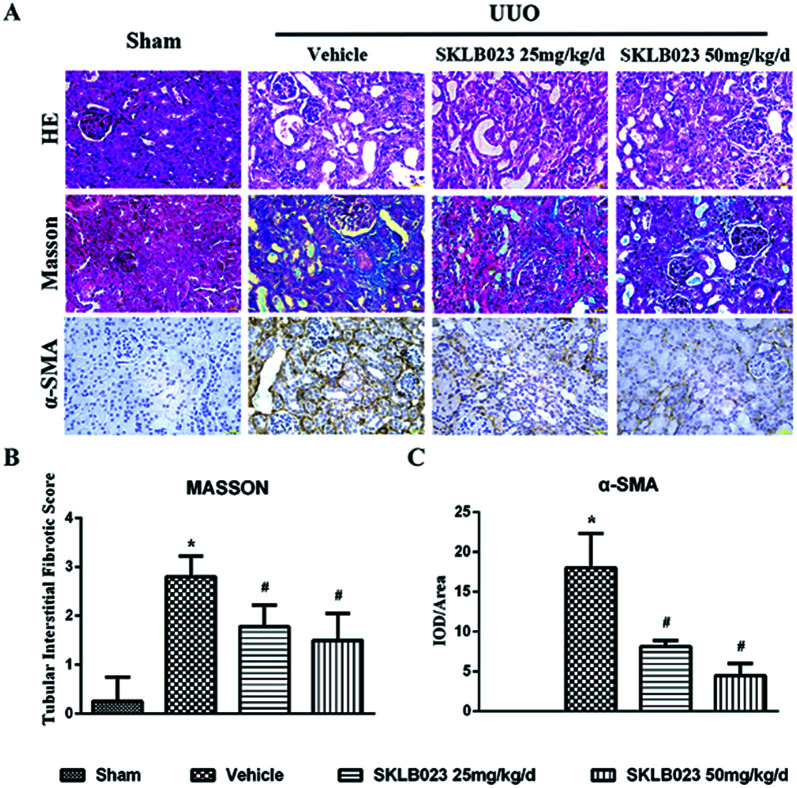
SKLB023 attenuates renal interstitial fibrosis in UUO. (A) Representative micrographs of hematoxylin and eosin (HE) and Masson's staining demonstrate kidney injury in indicated group; immunohistochemical staining of α-SMA protein in kidney tissue; (B) quantification of renal tubular interstitial fibrotic score (C) compared with the vehicle-treated-UUO group, the expression of α-SMA decreased in the UUO kidneys of SKLB023-treated groups. *P < 0.05 *versus* Sham, #*P* < 0.05 *versus* vehicle (*n* = 6).

We investigated the effect of SKLB023 on renal interstitial fibrosis in UUO, a representative model of renal interstitial fibrosis. As shown in Fig. 2, the UUO mice exhibited marked interstitial fibrosis in renal tissue by hematoxylin eosin (HE) and Masson's trichrome staining. Treatment with SKLB023 for 7 days dose-dependently reduced interstitial fibrosis score compared to that of UUO (Fig. 2B).

**Fig. 3:**
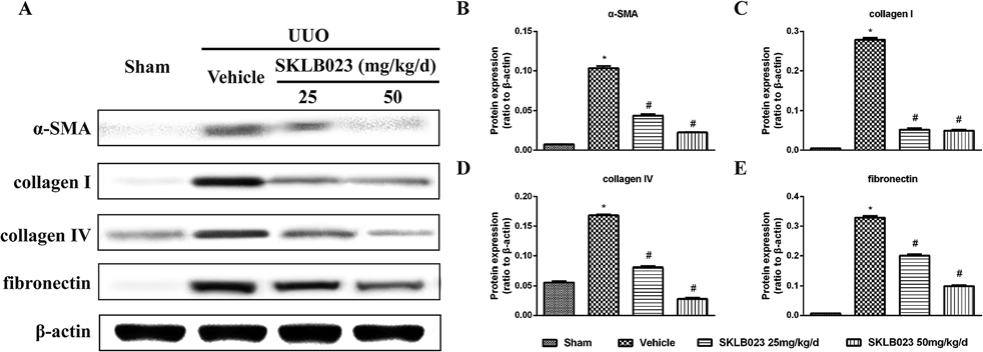
SKLB023 blocks on UUO-induced α-SMA, collagen I, collagen IV and fibronectin expression. (A) Kidney tissue lysates were subjected to immunoblot analysis with specific antibodies against α-SMA, collagen I, collagen IV and fibronectin. Expression levels of (B) α-SMA, (C) collagen I, (D) collagen IV and (E) fibronectin were quantified by densitometry and normalized with β-actin. *P < 0.05 *versus* Sham; #*P* < 0.05 *versus* vehicle (*n* = 6).

To investigate that SKLB023 suppressed *in vivo* myofibroblast activation and interstitial collagen fibrils, we examined the effect of SKLB023 on protein expression of α-SMA, col I, col IV and fibronectin in obstructive nephropathy. As exhibited in Fig. 2A–C, the results of the immunohistochemistry staining showed that renal expression of α-SMA in vehicle was more than that of Sham, and oral administration of SKLB023 reduced α-SMA expression in the kidney tissue of UUO. The Western blot analysis of whole kidney lysates indicated the increased expression of α-SMA, collagen I, collagen IV and fibronectin on day 7 after UUO injury, and SKLB023 treatment reduced the corresponding protein expression (Fig. 3). Importantly, SKLB023 at a dose of 50 mg per kg per day inhibited the fibrosis index more effective than that of 25 mg per kg per day (Fig. 3B–E), suggesting that treatment of SKLB023 prevented renal fibrosis in a dose-dependent manner.

### SKLB023 inhibits TGF-β1-induced activation of renal interstitial fibroblasts

**Fig. 4:**
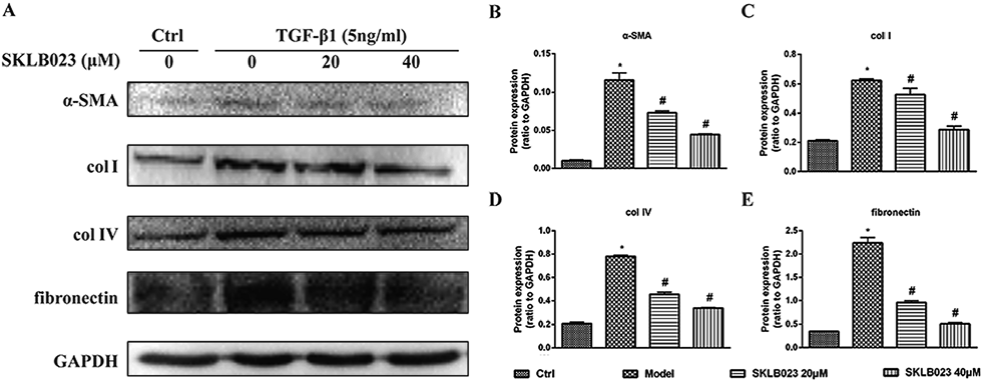
SKLB023 inhibits the expression of α-SMA, collagen I, collagen IV and fibronectin in TGF-β1 induced NRK-49F cells for 48 h. (A) Cell lysates were prepared and subject to immunoblot analysis with antibodies to α-SMA, collagen I, collagen IV and fibronectin. Expression levels of (B) α-SMA, (C) collagen I, (D) collagen IV and (E) fibronectin were quantified by densitometry and normalized with GAPDH. *P < 0.05 *versus* Ctrl; #*P* < 0.05 *versus* model. Data were expressed as means ± SD for three independent experiments.

To evaluate the regulatory effects of SKLB023 in TGF-β1-induced activation of renal interstitial fibroblasts, the expression of α-SMA, col I, col IV and fibronectin were examined by western blot analysis. In Fig. 4, the basal level of α-SMA, col I, col IV and fibronectin in serum starved rat kidney interstitial fibroblast cells (NRK-49F) were low under starved conditions. Following the treatment with TGF-β1, the expression levels of α-SMA, col I, col IV and fibronectin in NRK-49F cells were totally increased compared to that of control. Further, SKLB023 treatment significantly decreased the production of α-SMA, col I, col IV and fibronectin in a dose-dependent manner in NRK-49F cells compared with those of TGF-β1-induced group (Fig. 4B–E). Also in the 2D cell model that assessed by sirius red staining, SKLB023 significantly reduced the OD values of NRK-49F cells to exhibit anti-fibrotic activities (ESI Fig. 1†). These findings further confirmed the anti-fibrosis effect of SKLB023 in TGF-β1-induced NRK-49F cells.

### SKLB023 inhibits TGF-β1-induced Smad3 phosphorylation *in vitro*

**Fig. 5:**
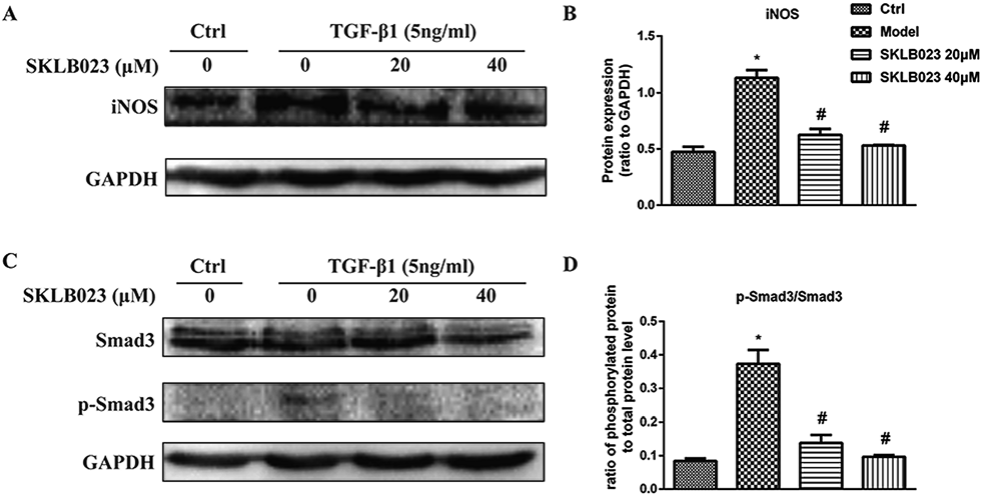
SKLB023 inhibits (A) and (B) the protein expression of iNOS (C) and (D) Smad3 phosphorylation in TGF-β1-induced NRK-49F cells for 48 h. *P < 0.05 *versus* Ctrl; #*P* < 0.05 *versus* model. Data were expressed as means ± SD for three independent experiments.

We also accessed the capacity of SKLB023 to inhibit iNOS expression by western blot analysis. As shown in Fig. 5A and B, the expression of iNOS protein were significantly inhibited by SKLB023 in a good dose-dependent manner.

Because TGF-β1 is a major cytokine that induces transformation of quiescent renal fibroblasts to myofibroblasts through the activation of Smad3,^22,23^ and to further explore the mechanism underlying antifibrotic effect of SKLB023, the TGF-β1-induced activation of the Smad3 pathway was evaluated in NRK-49F cells. As shown in Fig. 5C and D, the exposure to TGF-β1 resulted in the increase of Smad3 phosphorylation compared with that of control in NRK-49F cells. SKLB023 treatment dose-dependently attenuated the TGF-β1-induced Smad3 phosphorylation compared with that of TGF-β1-treated group.

### SKLB023 inhibits TGF-β1-induced Smad3 phosphorylation *in vivo*

**Fig. 6:**
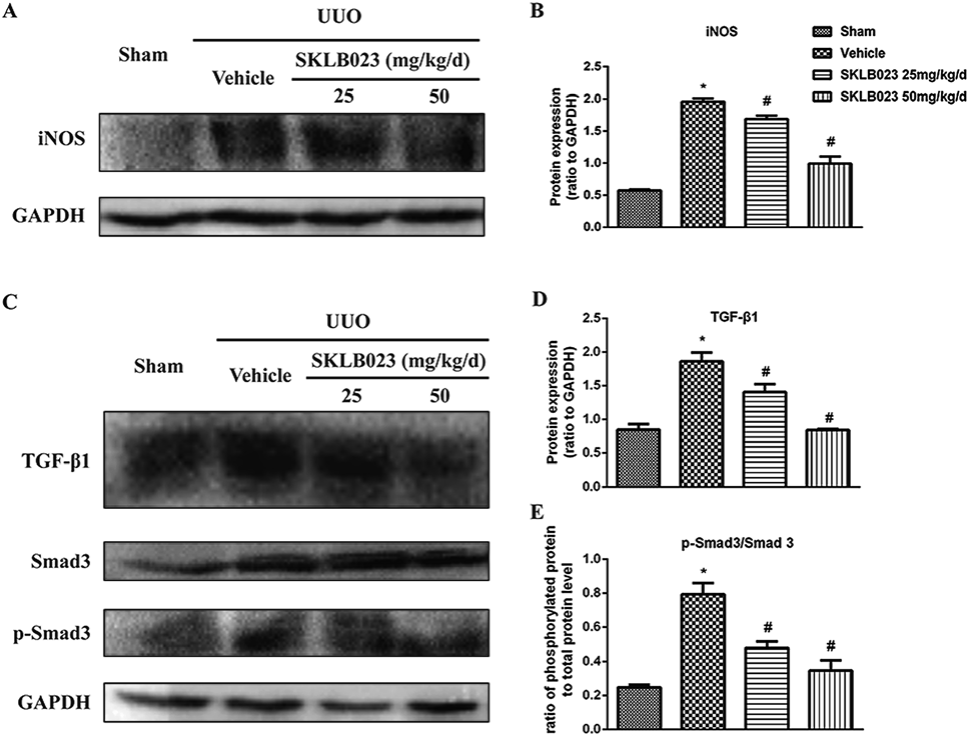
SKLB023 inhibits (A) and (B) the protein expression of iNOS (C)–(E) TGF-β1 and Smad3 phosphorylation in the obstructed kidneys. *P < 0.05 *versus* Sham; #*P* < 0.05 *versus* vehicle (*n* = 6).

We started with determining whether SKLB023 inhibited the expression of iNOS protein in UUO mice. In the control group, iNOS protein was low. After UUO surgery, the iNOS protein level was significantly increased compared with that of control group. Importantly, SKLB023 dose-dependently blocked the iNOS protein expression (Fig. 6A and B), stating that the down-regulation of iNOS protein by SKLB023 was favorable for the treatment of UUO in mice.

The increase in the expression of TGF-β has been considered as a major mechanism of renal fibrosis.^24,25^ As exhibited in Fig. 6C, ureteral obstruction markedly induced the expression of TGF-β1. Administration of different dosage of SKLB023 reduced the expression of TGF-β1 to 24% and 55%, respectively (Fig. 6D).

The TGF-β receptor activation triggers the phosphorylation and activation of downstream signaling mediators such as Smad3. Phosphorylated Smad3 subsequently translocated into the nucleus, where it controls the transcription of TGF-β response genes required for the development of fibrosis.^22^ Western blot analysis of kidney lysates indicated that expression of total Smad3 was not affected by UUO injury and SKLB023 (Fig. 6C). However, UUO injury-induced phosphorylation of Smad3 was abolished by oral administration of SKLB023 in a dose-dependent manner (Fig. 6C and E). Taken together, SKLB023 could inhibit TGF-β1/Smad3 pathways in UUO kidneys.

## Discussion

(*Z*)-*N*-(3-Chlorophenyl)-2-(4-((2,4-dioxothiazolidin-5-ylidene)methyl)phenoxy)acetamide (SKLB023), is a novel small-molecule iNOS inhibitor based on thiazolidine-2,4-dione moiety. By interfering TGF-β1/Smad3 signaling, our study first demonstrated that SKLB023 significantly inhibited renal interstitial fibrosis in UUO. Importantly, SKLB023 suppressed TGF-β1 and Smad3 phosphorylation *via* blocking the expression of iNOS. These results suggested that SKLB023 might be an effective therapeutic drug candidate for the treatment of renal interstitial fibrosis.

Renal interstitial fibrosis is the final common manifestation of various CKD, and progressive accumulation and deposition of ECM proteins in the interstitial area.^3^ In this study, the obstructed kidneys at 7 days after ureteral ligation culminated in various kinds of kidney damage, such as glomerulosclerosis, tubular apoptosis, and interstitial fibrosis. This change was consistent with other previous studies.^13,26–28^ The morphologic changes were accompanied by the increases in the expression of α-SMA, collagen, and fibronectin. To confirm the anti-fibrotic effect of SKLB023, we investigated the effect of SKLB023 on tubular interstitial fibrotic score, ECM deposition, collagen expression and fibroblast activation in UUO models. Due to the short half-life (*t*_1/2_ = 2.16 h),^29^ we designed daily administration to achieve its inhibitory effects. SKLB023 was reported that it was more effective with the increase of the dose in AIA models, therefore, we designed different dose of SKLB023 to test the inhibitory effects. As displayed in Fig. 3, oral administrations of SKLB023 at doses of 25 and 50 mg per kg per day inhibited α-SMA, col I, col IV and fibronectin and decreased the score of interstitial ECM deposition. Treatment with 50 mg per kg per day SKLB023 exhibited more inhibitory activity than dose of 25 mg per kg per day. These results suggested that SKLB023 attenuated fibrosis in a dose-dependent manner which might be a new candidate for treating renal fibrosis.

TGF-β1 is a well-known profibrotic cytokine in renal diseases and plays a critical role in renal fibrosis process. NRK-49F cells were normal rat kidney fibroblastic cells that could be the ideal cells *in vitro* experimental model. So, we used NRK-49F cells stimulated by TGF-β1 to address whether SKLB023 attenuated renal interstitial fibrosis. Our results showed that the exposure to TGF-β1 for 48 h increase the level of α-SMA, col I, col IV and fibronectin in NRK-49E cells. However, SKLB023 significantly reversed all above changes in a dose-dependent manner *in vitro*. We further used the sirius red staining two-dimensional (2D) model to quantify total collagen accumulation. This model could detect anti-fibrotic activities from selected compounds in previous studies.^30,31^ As shown in ESI Fig. 1,† treatment of SKLB023 markedly decreased size of the sirius red staining positive area. These results highlighted that SKLB023 prevented TGF-β1-mediated renal fibrosis *in vitro*.

Among the many fibrogenic factors that regulate renal fibrotic process, TGF-β is the key growth factor.^8^ TGF-β/Smad signaling participates in the process of fibrosis. TGF-β/Smad signaling through both canonical and non-canonical pathways to promote fibrosis. On the other hand, iNOS is crucial in fibrosis process.^13,32^ Previous studies have shown that melatonin prevents kidney damage by reducing expression of iNOS, and thereby suggested the harm of iNOS.^33^ Although its inhibition has been considered as a therapeutic strategy for several disease conditions,^21^ few studies have provided experimental evidence in the context of human fibroses diseases. One study investigated effect of oral administration of iNOS inhibitor FR260330 on liver fibrosis,^17^ and showed this inhibitor could improve liver fibrosis in rats by inhibiting TGF-β1 production. SKLB023 was designed for inhibiting iNOS. In our previous studies,^29^ we accessed the capacity of our targeted compounds contained thiazolidine-2,4-dione to inhibitory activity on iNOS. Twenty-two compounds derived from thiazolidine-2,4-dione moiety were evaluated. Among them, SKLB023 exhibited the most potent inhibitory effects (IC_50_ = 8.66 μM) without cytotoxicity on RAW 264.7 microphages shown by MTT assay (without or with LPS, IC_50_ > 100 μM). These results indicated the effectiveness and safety of SKLB023 in inhibiting iNOS.

It becomes clearer that we confirm the iNOS inhibitor SKLB023 could reduce fibrosis by decreasing the expression of TGF-β1 and its downstream molecules. Smad3 is the key downstream signaling mediators of TGF-β pathways, and its phosphorylation induced by interaction with TGF-β receptors has been recognized as a crucial step in TGF-β1/Smads signaling. Thus, we investigated whether SKLB023 could block TGF-β1-induced Smad3 activation *in vivo* and vitro models. Our results showed that SKLB023 inhibited the expression of TGF-β1, the *p*-Smad3/Smad3 in UUO model and in TGF-β1-induced NRK-49F cells for 48 h. Given that SKLB023 effectively inhibited the expression of iNOS, we tentatively proved that SKLB023 might be reasonable to inhibit TGF-β1/Smad3 signaling by reducing the overexpression of iNOS. Our study also demonstrated that SKLB023 could inhibit the process of renal interstitial fibrosis and maybe the potential effective drug candidate.

## Conclusions

In summary, SKLB023 as a potent small molecule compound was confirmed to inhibit renal interstitial fibrosis. SKLB023 suppressed the activation of TGF-β1/Smad3 signaling through blocking the expression of iNOS to attenuate interstitial fibrosis in UUO kidneys. These data suggested that SKLB023 was a novel therapeutic strategy for antifibrotic intervention.

## Conflicts of interest

There are no conflicts to declare.

## Supplementary Material

RA-008-C8RA00018B-s001
