# Supplementary material for: SKLB023 hinders renal interstitial fibrosis in obstructive nephropathy by interfering TGF-β1/Smad3 signaling
Source: RSC Adv. 2018 Feb 6;8(11):5891–6. doi: 10.1039/c8ra00018b (PMC9078199; doi:10.1039/c8ra00018b)
Supplement: RA-008-C8RA00018B-s001 [file RA-008-C8RA00018B-s001.pdf]

Electronic Supplementary Material (ESI) for RSC Advances.
This journal is © The Royal Society of Chemistry 2018

SKLB023 Hinders Renal Interstitial Fibrosis in Obstructive Nephropathy by interfering TGF-β1/Smad3 signaling

Yanhuan Feng [1], Jun Xu [1], Fan Guo[1], Rongshuang Huang, Min Shi, Lingzhi Li, Liang Ma*, and Ping Fu*

Kidney Research Institute, Division of Nephrology, West China Hospital of Sichuan University, Chengdu 610041, China.

[1] The authors contributed equally to the work.

*Correspondence: Kidney Research Institute, Division of Nephrology, West China Hospital of Sichuan University, No. 37 Guoxue allay, Chengdu 610041, China. Tel.: +86 28 85164167, Fax: +86 28 85423341. E-mail address: liang_m@scu.edu.cn (L. Ma), fupinghx@scu.edu.cn (P. Fu).

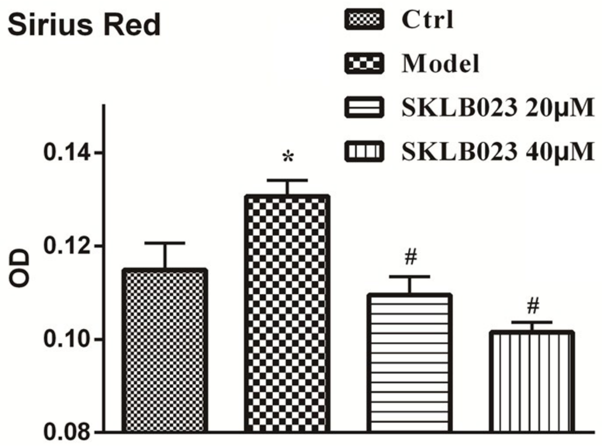

Supplemental Figure 1. SKLB023 inhibited TGF-β1-induced total collagen accumulation in NRK-49F cells. Cells cultured in 96-well plates were treated with increasing doses of SKLB023 (0-40 μM) with 5 ng/ml TGF-β1 for 48 h. Total collagen accumulation was stained by Sirius Red staining and quantified by spectrophotometric analysis. *P<0.05 versus Ctrl; #P<0.05 versus Model. Data were expressed as means ± SD for three independent experiments.
